# Blood Cultures in the Early Diagnosis of Enteric Fever

**Published:** 1909-02-27

**Authors:** 


### Medicine.

### BLOOD CULTURES IN THE EARLY DIAGNOSIS OF ENTERIC FEVER.

Valuable as Widal's test is in the diagnosis of typhoid fever it is not so perfect that one can afford to rely upon it alone. It may be ten days or more from the onset before the Widal reaction is positive, and under present conditions one may be obliged to remain in doubt as to the nature of the disease all that time.

An additional means of arriving at the diagnosis is by blood-culture; for quite early in enteric fever it has been shown that typhoid bacilli are to be found in the blood. Indeed, there is considerable evidence to show that they are most numerous there in the earlier stages of the disease, becoming fewer later on. The bacilli may be present before the Widal reaction is positive; whilst by the time the latter develops blood-culture may discover no bacilli. In other words, in any case of suspected typhoid fever in which the serum as yet gives no clumping reaction it would seem to be wise to resort to blood-cultures by some such method as that employed by Major S. E. B. Statham, R.A.M.C.

The patient's finger is sterilised by the application of a wet dressing such as carbolic lotion 1 in 20, or perchloride of mercury solution 1 in 500 an hour or two before the culture is to be taken. The antiseptic may be removed by sterilised water. The pulp of the finger is pricked with a broad-bladed needle, the blood collected in a sterile pipette, and transferred to a small test tube containing sterile ox bile. By the intermittent application of a tourniquet to the patient's finger, and, if necessary, by a second prick or two, it is comparatively easy to obtain one cubic centimetre or more of blood. The admixture of the latter with bile in the test tube is incubated for sixteen hours, and a drop of the incubated mixture smeared with a glass spreader over an agar plate. After a further sixteen hours' incubation the plates are ready to examine.

Out of seventy-two cultures from seventy-two different patients organisms of the typhoid-colon group were isolated by Major Statham in forty-seven, that is to say 65.3 per cent., or very nearly two-thirds of all cases. When the reason for the success of the method in some cases and its failure in others was investigated interesting confirmation was discovered of the theory that typhoid bacilli enter the blood stream at a very early stage of typhoid fever and diminish greatly or even disappear from it towards the end of the febrile period. When the period of the fever was calculated in each case from two fixed points, namely, the date on which the temperature subsequently became normal, and the date on which the blood-culture was taken, it became clear that in patients in whom more than twenty days of fever remained after the culture was taken a positive result was obtained in every case; that when from fourteen to twenty days of fever remained after the blood-culture, successful results were obtained in fifteen out of sixteen cases; that when ten to fourteen days of fever remained after the culture was taken growths of the typhoid-colon group were obtained in ten out of fourteen cases; that in those cases in which the patients suffered only seven to ten more days of fever after the culture had been taken positive results were obtained in four out of nine cases, or 44 per cent.; whilst when fewer than seven days of fever remained after the blood culture positive results were obtained in two only out of seventeen cases, *i.e.* 12 per cent.

It is, therefore just during those first ten days of the fever when Widal's reaction has not yet become positive that the blood-cultures are most likely to be obtained. When both a blood culture and a Widal's test were carried out within three or four days of each other, as a rule only one of the two tests was successful.

The general conclusions that can be drawn from Major Statham's work seem to be that even when such small amounts of blood as one cubic centimetre are used for purposes of culture, positive results can be obtained in over 90 per cent. of typhoid cases if the culture is taken within ten days of the onset of the disease.

As this small quantity of blood can readily be obtained from the patient's finger pulp, and further as the use of bile as a culture medium is simple and easy of application, the method should be one of great value in the early diagnosis of typhoid fever. It would seem, therefore, that, by carrying out both a culture from the blood of the patient and a serum test, few cases of typhoid fever would escape diagnosis within thirty-six hours.

